# Reinforcement of Epoxy Composites with Application of Finely-ground Ochre and Electrophysical Method of the Composition Modification

**DOI:** 10.3390/polym12071437

**Published:** 2020-06-27

**Authors:** Amirbek Bekeshev, Anton Mostovoy, Lyazzat Tastanova, Yulia Kadykova, Svetlana Kalganova, Marina Lopukhova

**Affiliations:** 1 K. Zhubanov Aktobe Regional State University, Aliya Moldagulova Avenue 34, 030000 Aktobe, Kazakhstan; amirbek2401@gmail.com (A.B.); lyazzatt@mail.ru (L.T.); 2 Yuri Gagarin State Technical University of Saratov, Polytechnichskaya St., 77, 410054 Saratov, Russia; Kadykova06@yandex.ru (Y.K.); sgkalganova@sstu.ru (S.K.); mlopuhova@yandex.ru (M.L.)

**Keywords:** Epoxy resin, modification, filler, ocher, microwave modification

## Abstract

The conducted studies have proven the possibility of the directed control of operational properties of epoxy composites, due to the addition of finely-ground ocher into their composition, and the use of microwave modification of the epoxy composition. The rational content of ocher as a modifying additive (0.5 parts by mass) and a filler (75 parts by mass) of the epoxy composition has been selected, which ensures the improvement of the studied complex of physical-mechanical properties. It has been proven that ocher affects the structure formation processes and the structure of the epoxy composite, thus increasing its thermal, heat and fire resistance. During the research, the application efficiency has been proven, and the optimal parameters of the microwave modification (power—350 W; duration—30 s) of epoxy compositions filled with ocher, which increase physical-mechanical characteristics of composites, have been selected.

## 1. Introduction

One of the promising branches of chemical industry is the production of polymer materials [1]. At present, materials based on cross-linked polymers play an important role in various fields of human activity due to their structure, which gives the materials a number of valuable qualities, such as high hardness, chemo- and heat resistance, etc. In terms of the production volume and the degree of consumption, epoxy binders occupy an important place in global industry, and are widely used in various sectors of consumption [1,2,3,4,5,6]. At the same time, they have a number of negative qualities: increased fragility, fire hazard, low resistance to climate factors [4,5,6,7,8,9].

One of the main directions of the further development of epoxy composites with enhanced operational properties is the addition of various fillers (basalt, talc, chromite, metal oxides, technogenic waste from industrial enterprises) into their composition, which can significantly decrease the cost of finished products by reducing the cost of expensive binders, as well as give them various functional properties [2,3,4,5,6,7,8,9,10,11,12].

Technogenic waste from industrial enterprises is often used as dispersed fillers of epoxy resins. The authors of [13,14] propose to use fly ash, which is a by-product of coal burning at thermal power plants, as a filler for epoxy composites. The filler content ranged from 10 to 50 vol %. The conducted studies have showed that, when adding fly ash into the epoxy composite, the tensile strength increases, as the filler amount increases to a critical point (30 vol %), and then decreases significantly. On the other hand, the compressive strength of the composite continuously increased with the increase in the amount of fly ash. In addition, it was found that the reduction of fly ash particle size provides the production of epoxy composites with higher physical and mechanical properties.

In [15], marble processing wastes obtained from wastewater using various coagulants, such as sepiolite, zeolite or pumice, were used as a filler for epoxy composites. The results showed that marble waste with all coagulants can significantly improve the heat resistance of the epoxy matrix at temperatures above 350 °C. Moreover, the addition of this filler provides an increase in the surface hardness and tensile strength, in comparison with the pristine epoxy matrix.

The effectiveness of using brick dust as a cheap, active filler of an epoxy polymer, which provides an increase in both physicochemical and mechanical properties, was proved in [16,17]. It was found that the addition of 30–50% by weight of brick dust has an effect on the processes of the structure formation and the structure of the epoxy composite, thus providing an increase in bending and compression strength, as well as a significant increase in impact strength, in comparison with an unfilled epoxy composite.

In recent decades, composites reinforced with natural fibers have aroused wide research and engineering interest, due to their low density, high specific strength, low cost, light weight, processability and biodegradability. In the article [18], wood dust was used to increase the physicomechanical characteristics of epoxy composites. Studies have shown that the addition of wood dust into the epoxy composite increases the tensile and bending strength to a certain filler content, and then gradually decreases. The optimal content of wood dust in the epoxy composition is 10–15%, which provides the maximum physical and mechanical characteristics are achieved.

As nanofillers, graphen, carbon nanotubes, fullerenes, astralenes, as well as titanium and silicon dioxide, diamond batch, white carbon black, zinc borate nanoparticles, etc., are widely used [3,11,19,20,21,22,23,24,25,26,27].

The authors [23,24] proposed a technology for combining an epoxy oligomer with a nanoscale filler, which consists of joint grinding using microwave-assisted ball milling. Such a modification ensures the chemical interaction of the epoxy oligomer with a titanium hydride, proved by IR spectroscopy, which could not be obtained by conventional grinding using a ball mill. The proposed technology leads to improved compatibility of TiH_2_ nanoparticles with the epoxy oligomer, their uniform distribution in the composition, which provides an increase in the physicomechanical characteristics of polymer composite materials.

In Ref [25], zinc borate nanoparticles coated with a silicon oxide were obtained to create fire-resistant composites. It was found that zinc borate nanoparticles modified with a silicon oxide are more uniformly distributed in the epoxy resin, compared to the unmodified additive.

Despite the enormous number of works devoted to the study of the effects of various fillers, there are still insufficiently studied issues related to the influence of fillers on the structure formation processes, the structure and performance characteristics of polymer composite materials, which predetermines the direction of the research of this work.

Currently, to intensify the processes of modification of polymer materials, electro-physical methods are widely used, such as elastic vibrations of sound and ultrasonic frequency ranges, vibration processing, high-frequency currents, laser, electronic and ultraviolet radiation. The need for alternative technologies for the modification of polymers is connected with the multistage nature of traditional processes, high energy and labor costs, and environmental tensions in production [28,29,30].

Studies on the use of electro-physical methods of processing materials and products have shown the efficiency of using the microwave energy for this purpose. Microwave technologies, in contrast to traditional modification methods, have several advantages: they make it possible to reduce the duration of technological processes, to simplify the production installation, to lower energy consumption, to improve the environmental condition and cleanliness of production, to obtain products of a new, better quality. They also provide volumetric and inertia-free heating, the possibility of forming and maintaining the required distribution of the temperature field in any finite region of space, etc. [28,29,30]. In this regard, in this work microwave modification was used to improve deformation-strength properties of epoxy composites.

The aim of this work is to improve physicochemical and deformation-strength properties of epoxy-based composites by adding a polyfunctional modifier of oligo(resorcinophenyl phosphate) with terminal phenyl groups Fyrolflex (ORPP) and a dispersed mineral filler—ocher.

In this article has studied the effect of adding the mineral filler on the mechanical properties, the curing process, physicochemical properties of epoxy composites and the effects of using microwave technology on the mechanical properties of ocher/epoxy composites. This work shows an important reference value for modification, optimizing and designing of epoxy compositions for the production of high-performance ocher/epoxy composites.

## 2. Materials and Methods

### 2.1. Materials

As a polymer matrix, epoxy resin ED-20 (GOST 10587-93) was used, which is characterized by a low viscosity—13–20 Pa·s; a narrow limit of the epoxy groups content—20.0–22.5%; stability of physico-chemical properties, epoxy equivalent—195–216 g/mol, manufactured by CHIMEX Limited (St. Petersburg, Russia). 

As a hardener of an epoxy oligomer, an amine type hardener was used—polyethylene polyamine (PEPA) (TS 6-02-594-85), manufactured by CHIMEX Limited (St. Petersburg, Russia). It is capable of forming a three-dimensional network structure without heating, its molecular mass is 230–250 g/mol, its amine number is 1250 mg KOH/g and its viscosity is 0.6–0.9 Pa·s.

To plasticize epoxy composites, we used oligo(resorcinophenyl phosphate) with terminal phenyl groups (ORPP), with a purity of 99%, manufactured by ICL Industrial Products America Inc. (St. Louis, MO, USA). The choice of ORPP is due to the presence of combustion inhibitors—phosphorus (10.7%). When the composite thermally decomposes, the presence of phosphorus ensures an increase in the yield of carbonized structures. These structures are a physical barrier for the inter-diffusion of the oxidant and combustible gases to the combustion zone, which, in general, reduces the flammability of epoxy composite [9,12,20].

There are wide possibilities for improving the characteristics of composite materials by using both plasticizers and cheap and effective fillers [2,3,4,5,6,8,9,10,11,12], including dispersed mineral fillers, e.g., finely dispersed ocher. We took ocher from the Priorskoe deposit (Khromtau raion, Aktobe oblast, Kazakhstan) with the particle size of ≤40 μm.

### 2.2. Preparation of Epoxy Composites

A previously developed composition, consisting of 100 parts by mass of epoxy resin brand ED-20, 40 parts by mass of ORPP and 15 parts by mass of a PEPA hardener was used as a polymer matrix [9,12].

Ocher was introduced into ORPP-modified epoxy compound as a modifying additive (0.1–1.0 wt. part per 100 wt. parts of the compound) or filler (10–100 wt. parts). To ensure more uniform distribution of ocher and to activate its surface and the binder, the formulation was subjected to ultrasonic treatment at 22 ± 2 kHz for 60 min. The mixture was degassed at 25 ± 5 °C for 30 min under vacuum, before curing.

**Figure 1:**
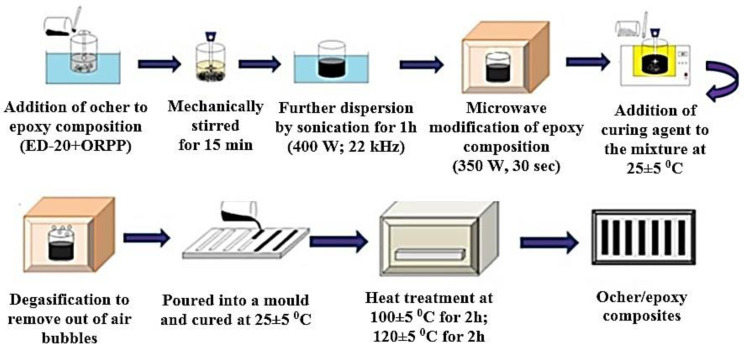
Schematic illustration for the preparation process of ocher/epoxy composites.

The preparation process of ocher/epoxy composites is shown in Figure 1.

### 2.3. Characterization of Finely Ground Ochre

The research was carried out using the following methods: the study of the surface morphology of ocher was carried out using a Tescan VEGA 3 SBH scanning electron microscope (Brno, Czech Repuplic); X-ray phase analysis was performed using ARL X’TRA X-ray diffractometer (CuK_α_ radiation, λ = 0.15412 nm, angle range 2θ 5°–60°); the diffraction patterns were interpreted using Powder Diffraction File-2 (PDF-2) database of the International Center for Diffraction Data (ICDD) and the Crystallographic Search-Match program, version 3.1.0.2.B; determination of the particle-size distribution in the interval 0.01–1000 μm by laser diffraction with a Fritsch Analysette-22 Nanotech analyzer (Fritsch, Germany), using water as a dispersion medium; determination of the specific surface area of the samples with a Quantachrome Nova 2200 surface area and porosity analyzer (Boynton Beach, FL, USA) from the low-temperature nitrogen adsorption.

### 2.4. Testing of the Composites

**Figure 2:**
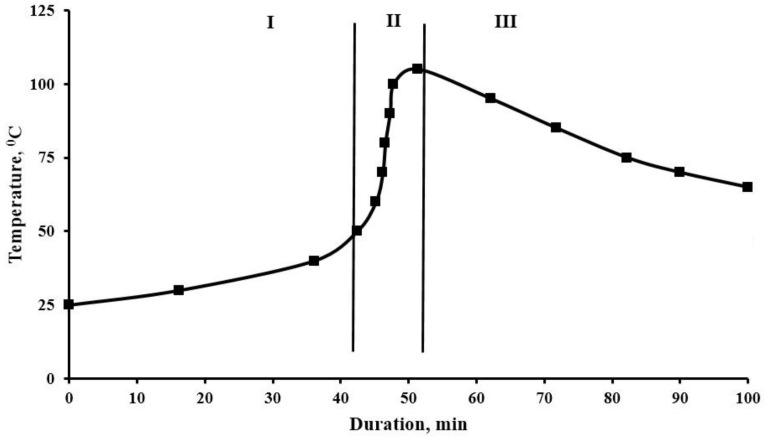
Kinetics of the epoxy composition curing.

Mechanical properties of composites were investigated by the universal testing machine ‘‘WDW-5E’’ (Time Group Inc., Beijing, China) at a 20 mm/min loading rate. The testing accuracy was 1%. The bending stress and flexural modulus was determined according to [ISO 178: 2010], the tests were carried out on samples in the form of blocks with 4 mm thickness, 10 mm width and a working part length of 80 mm. The strength and modulus of tensile elasticity was determined according to [ISO 527-2: 2012], the tests were carried out on samples in the form of spatulas with 4 mm thickness, 10 mm width and a working part length of 50 mm. The compressive strength was determined according to [ISO 604: 2002], the tests were carried out on samples in the form of cube with rib length 30 mm. The impact strength was determined according to [ISO 179-1: 2010], using an impact testing machine LCT-50D (Beijing United Test Co., Ltd., Beijing, China). The heat resistance, according to Vicat, was determined according to [ISO 306: 2004], method B50-load 50 N; the rate of temperature increase was 50 °C/h. The change in mass, rate of change in mass and magnitude of thermal effects during the heating of the samples was studied using the method of thermogravimetric analysis, with the help of a derivator of the “Paulik–Paulik–Erdei” system of the MOM brand Q-1500D (Budapest, Hungary), under the experimental conditions: weight—100 mg, medium—air, heating interval—up to 800 °C, heating rate—10 °C/min, relative error does not exceed 1%. The study of the surface morphology of the samples was carried out using a Tescan VEGA 3 SBH scanning electron microscope (Brno, Czech Repuplic). We have studied the process of the composition curing with the construction of the dependence G = f (τ, T), where τ is the duration of the process in minutes; T—temperature, °C. In this analysis, the thermal insulation of objects was provided. It was possible to study the effect of heat dissipation on the curing process. The temperature rise curve consists of three characteristic sections (Figure 2).

The first section characterizes the viability of the composition before the start of temperature rise; the second section of the rapid increase in temperature up to the inflection point characterizes the self-accelerating curing reaction; in the third section of the curve, the chain growth rate decreases after the inflection point. Then, the sample cooling begins. The inflection point on the curve of the curing temperature versus time, which characterizes the maximum curing rate, can most accurately be determined as the maximum value of the derivative of the function of temperature change in time in sections I and II, Figure 2. To do this, we find the equation of the best power polynomial approximating the line for the experimental data by the method of least squares. Using these curves, we determine the parameters of the curing process [8,9].

## 3. Results and Discussion

There is a great potential for improving the characteristics of composite materials, which is due to the use of both plasticizers and inexpensive and effective fillers, which include dispersed mineral fillers, in particular ground ocher, the chemical composition of which is presented in Table 1.

As a result of the research, it has been found that ocher mainly consists of iron oxides, aluminum oxide, chromium and nickel, and it also contains small impurities of Ti, Cu, Ca oxides.

**Figure 3:**
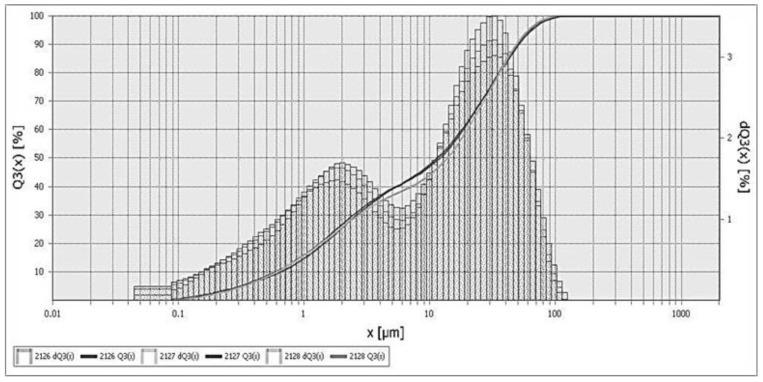
Fractional composition of ocher.

**Figure 4:**
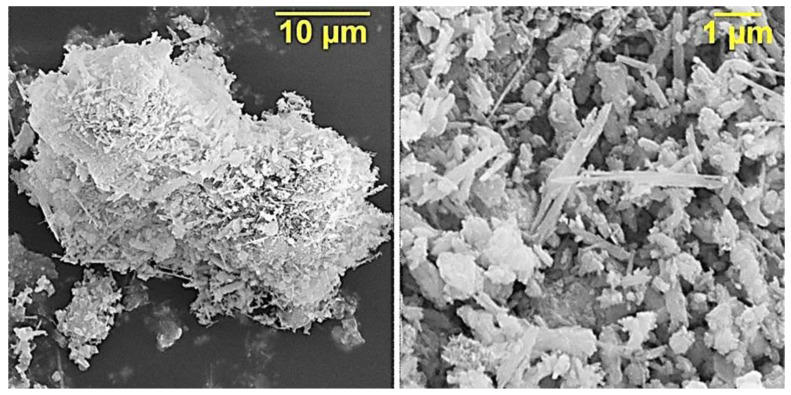
SEM data of ocher particles.

The fractional composition of ocher is characterized by a bimodal distribution and is represented by particles from 0.1 to 100 μm, with average particle sizes of 2–3 μm and 30–40 μm (Figure 3), which is also confirmed by SEM data (Figure 4).

**Figure 5:**
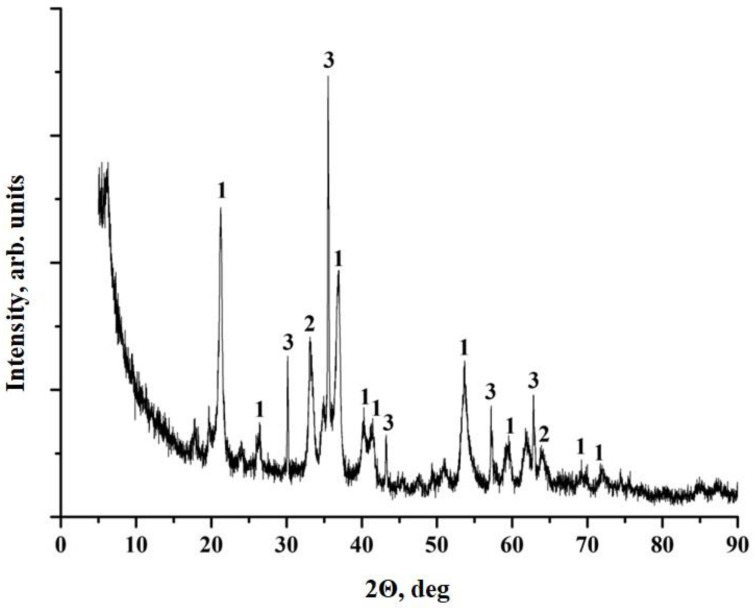
XRF pattern of ocher particles: 1—FeO(OH) goethite; 2—Fe_2_O_3_ hematite; 3—Fe_3_O_4_ magnetite.

According to XRF data, ocher is represented by three main structures: goethite (FeO (OH)), hematite (Fe_2_O_3_) and magnetite (Fe_3_O_4_) (Figure 5).

Goethite has needle-shaped crystals, hematite crystals are flattened, from tabular to scaly or lamellar, magnetite is represented by cubic crystals. In addition, magnetite has strong magnetic properties, which imparts magnetic properties to composite materials, and thereby expands the possible areas of their application.

Due to the fact that ocher particles have a scaly and needle-shaped form, we can attribute ocher to active reinforcing fillers. The presence of needle-shaped particles must provide the effect of micro-reinforcement, which will lead to the improvement of physico-mechanical properties of composites based on it.

The specific surface area of chromite particles, determined on the specific surface area and porosity analyzer Quantachrome Nova 2200 using a low-temperature nitrogen adsorption method, is equal to 7.2 m^2^/g.

Thus, the analysis of the structure, chemical composition and specific surface of finely ground ocher has shown that it can be used as a structuring additive and a filler for epoxy composites, which should improve their operational properties. In this case, preliminary drying of the filler is required, since the moisture content in ocher amounts to 27%. Drying was carried out at 105 °C for 3 h to obtain constant mass.

The conducted studies have shown that the most rational addition of ocher as a modifying additive is 0.5 parts by mass, since this ensures higher values of the studied physical and mechanical properties: bending stress increases by 30% and flexural modulus increases by 58%, strength increases by 75% and tensile modulus increases by 20%, impact strength increases by 83% (Table 2).

The structuring effect of ocher is observed in the effect of small additives. All known polymers are microheterogeneous, therefore, they contain both tightly packed (ordered areas) and loose (more defective zones), in which small modifier additives are concentrated. They play an essential structural-modifying role, contribute to kinetically stimulated polymer ordering, and increase the mobility of the loop chains, ensuring their denser packing [7,8,12].

**Figure 6:**
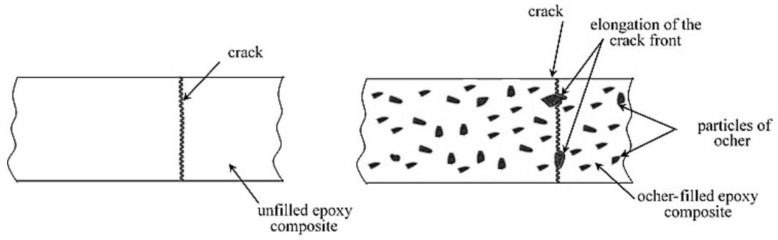
Model for the role of ocher as modifying additive in the epoxy composite.

In terms of energy concept, the hardening of ocher-filled epoxy compositions occurs due to the increase in the energy required to fracture the material, which is associated with the elongation of the crack front, due to the flow of particles around the crack, Figure 6. The decrease in strength at a lower or higher optimum filler content is the result of an inefficient interaction of the polymer matrix with filler particles. At low degrees of filling (0.1 parts by mass), the particles are relatively far removed from each other in the volume of the composite, therefore, the effect of crack delaying is significantly reduced. At higher degrees of filling (1 part by mass), the particles are packed more densely, and the composite is a continuous medium, in which the crack front ceases to interact with individual particles [12].

In terms of reducing the cost of production, the addition of ocher as a filler of the epoxy composite (50–100 parts by mass) is effective, while the addition of 75 parts by mass is the most rational, since the physico-mechanical characteristics are improved: bending stress increases by 27% and modulus of elasticity in bending increases 4.5 times; strength increases by 50% and the tensile modulus increases two times; resistance to impact loads increases by 50%; and we also observe that breaking stress in compression increases by 56%, compared to an unfilled plasticized epoxy composite (Table 2).

According to published data [1,12], at high degrees of filling (75 parts by weight), an increase in strength is explained by the fact that, in the presence of a filler, neighboring ocher particles crosslink in the structure of the epoxy composite by polymer macromolecules. As a result, the bridges of individual macromolecule chains appear between the filler particles, which helps to limit the number of possible conformations of macromolecules and their segment motion, which leads to an increase in the elastic modulus and strength of the composite.

**Figure 7:**
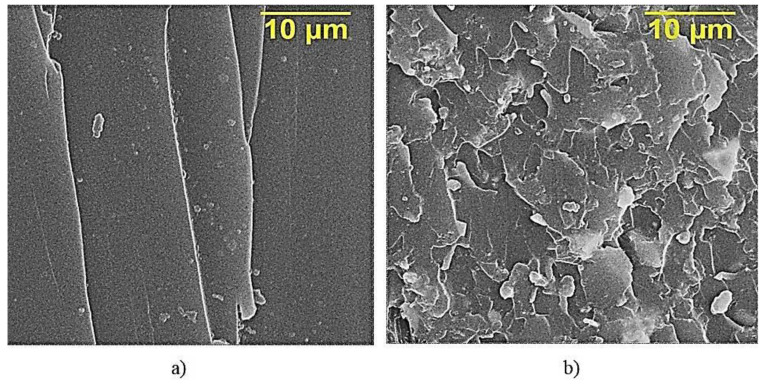
SEM of the surface of the destruction of epoxy composites: (**a**)—without ocher; (**b**)—with 75 parts by mass of ocher, relative to ED-20.

Figure 7a presents fractography of the destruction of epoxy composite samples without ocher, which is characterized by a rather smooth fracture surface, showing a low ability to crack resistance [31]. When ocher was added into the epoxy composition, they affect the morphology of the matrix—layered structures appear (Figure 7b).

In addition, under the action of ultrasound, it is possible to evenly distribute the filler in the epoxy composition, and to avoid its aggregation.

When estimating the effect of the modifying additive on network polymers, we should take into account the fact that the curing process takes place in the presence of a developed surface of the solid material (ocher), thus affecting the kinetic characteristics of the polymerization reaction during curing, as well as the formation of the phase structure of the material [7,8,12]. We should also note a significant role of the adsorption interaction of the components of the oligomeric composition with the solid surface of the ocher.

**Figure 8:**
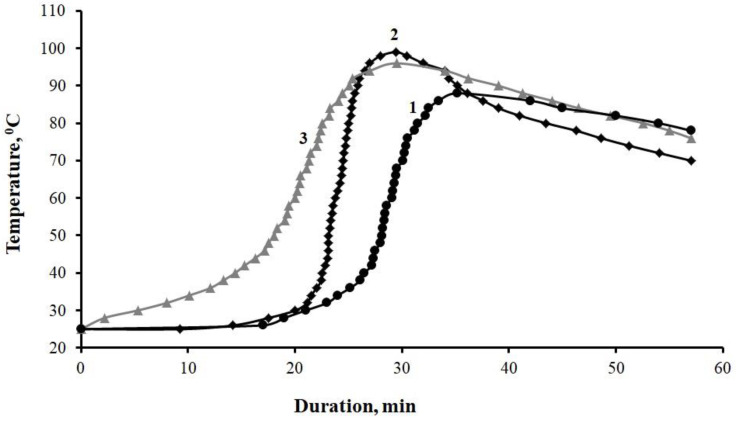
Kinetic curves of the curing process of compositions, parts by mass: 1—100ED-20 + 40ORPP + 15PEPA; 2—100ED-20 + 40ORPP + 0.5Ocher + 15PEPA; 3—100ED-20 + 40ORPP + 75Ocher + 15PEPA.

The analysis of the curing kinetics of epoxy compositions has shown that the addition of ocher affects the structure formation processes (Figure 8), which can be seen in the reduction of the gelation process duration from 27 to 17–21 min, and the curing process duration from 38 to 29–30 min, maximum curing temperature increasing from 88 °C to 96–99 °C (Table 3).

Due to the fact that ocher itself is a heat-resistant material, its addition to an epoxy composite leads to an increase in Vicat heat resistance from 132 °C to 148–210 °C, Table 4.

The addition of ocher into an epoxy composite provides an increase in the composite heat resistance, which can be seen in a shift in the initial temperature of the main stage of destruction to higher temperatures (from 230 °C to 240–245 °C), while the yield of carbonized structures also increases from 54% to 58–76%, providing a decrease in the emission of volatile pyrolysis products into the gas phase, which reduces flammability of the epoxy composite. With the addition of ocher, an increase in the flammability indicator oxygen index from 28 to 32% by volume is observed (Table 4).

**Figure 9:**
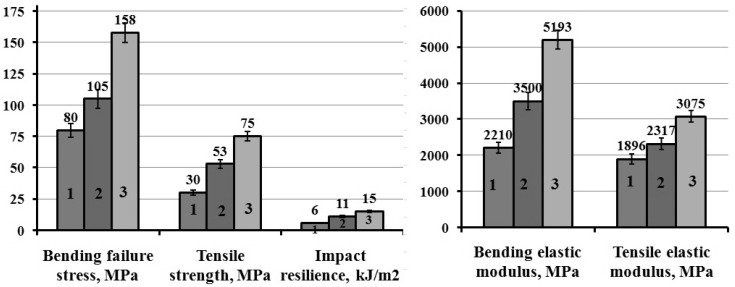
Physico-mechanical characteristics of epoxy composites: 1—epoxy composite, without filler and microwave modification; 2—epoxy composite with 0.1 parts by mass of ocher relative to ED-20, without microwave modification of composition; 3—epoxy composite with 0.1 parts by mass of ocher relative to ED-20, and after microwave modification of composition.

Microwave modification was used to improve deformation-strength properties of epoxy composites. The uncured hardener-free epoxy composition was subjected to microwave modifications after ultrasonic dispersion of the components, then a hardener was added, and the curing process was carried out under normal conditions at room temperature. The studies made it possible to determine the most rational mode of microwave processing: power—350 W, duration—30 s (Table 5), which ensured the maximum physical and mechanical characteristics: composite bending strength increased by 26%, tensile strength increased by 29% and toughness increased by 18% (Figure 9).

**Figure 10:**
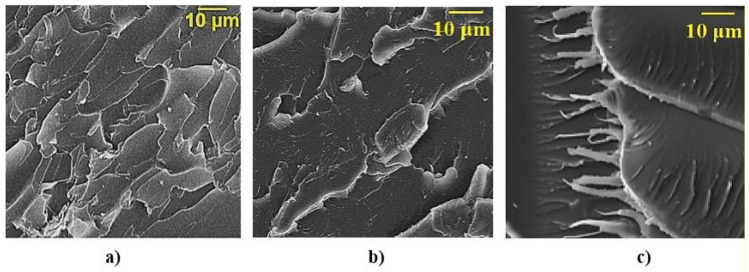
SEM of the surface of the destruction of epoxy composites: (**a**)—without microwave modification; (**b**,**c**)—after microwave modification.

Thus, the studies have shown the effectiveness of applying microwave modifications of epoxy compositions filled with ocher, which ensures the improvement of physico-mechanical characteristics of composites, which is explained by additional structuring of the material as a result of microwave processing. In Figure 10b,c, the destruction surface of an epoxy composite after the microwave modifications of epoxy compositions can be seen. A very large fraction of plastic destruction appears in the sample, the borders of the scales become blurred, and their size increases. This may be on account of the formation of a dense cross-linking network around the ocher particles and the oligomer epoxy groups.

## 4. Conclusions

As a result of these studies, the effectiveness of using ocher as a cheap, active filler of an epoxy polymer has been proven, which provides the improvement of both physicochemical and mechanical properties of epoxy composites. The rational content of ocher as a modifying additive (0.5 parts by mass) and filler (75 parts by mass) of the epoxy composition has been selected, which ensures the improvement of the studied complex of physic-mechanical properties. It has been proven that ocher affects the structure formation processes of the epoxy composition, which can be seen in the reduced duration of the gelation process from 27 min to 17–21 min, and the duration of the curing process from 38 min to 29–30 min, with an increase in the maximum curing temperature from 88 °C to 96–99 °C. It has been proven that the addition of ocher into the epoxy composite provides an increase in the thermal stability of the composite, which can be seen in a shift in the initial temperature of the main stage of destruction to higher temperatures (from 230 °C to 240–245 °C). We can also see an increase in the yield of carbonized structures from 54% to 58–76%, providing a decrease in the emission of volatile pyrolysis products into the gas phase, which ensures a decrease in the flammability of the epoxy composite.

The efficiency of the application has been proven, and the optimal parameters of the microwave modification of epoxy compositions filled with ocher have been selected, which increase the physico-mechanical characteristics of composites.

## Figures and Tables

**Table 1:** Chemical composition of ocher.

Component	Concentration, %
FeO(OH) Fe_2_O_3_ Fe_3_O_4_	85.95
Al_2_O_3_	4.63
Cr_2_O_3_	2.80
SiO_2_	2.31
NiO	2.88
TiO_2_	0.38
CuO	0.66
CaO	0.24
S	0.08
P	0.07

**Table 2:** Properties of epoxy composites.

The Composition, Parts by Mass Cured by 15 Parts by Mass of PEPA	G_ben_, MPa	E_ben_, MPa	G_com_, MPa	G_ten_, MPa	E_ten_, MPa	a_im_, kJ/m^2^
100 ED-20 + 40 ORPP	80 ± 3.2	2210 ± 88	100 ± 4.0	30 ± 1.5	1896 ± 75	6 ± 0.3
100 ED-20 + 40 ORPP + 0.1 ocher	87 ± 3.5	3206 ± 120	102 ± 4.1	41 ± 2.0	2113 ± 84	7 ± 0.3
100 ED-20 + 40 ORPP + 0.5 ocher	105 ± 4.2	3500 ± 140	105 ± 4.1	53 ± 2.6	2317 ± 92	11 ± 0.5
100 ED-20 + 40 ORPP + 1.0 ocher	75 ± 3.0	4073 ± 152	110 ± 4.4	43 ± 2.2	3040 ± 121	5 ± 0.2
100 ED-20 + 40 ORPP + 10 ocher	80 ± 3.2	4373 ± 164	114 ± 4.5	53 ± 2.6	3242 ± 122	5 ± 0.2
100 ED-20 + 40 ORPP + 30 ocher	76 ± 2.8	5568 ± 220	125 ± 5.0	36 ± 1.8	3512 ± 140	7 ± 0.3
100 ED-20 + 40 ORPP + 50 ocher	74 ± 2.2	7462 ± 240	145 ± 5.6	41 ± 2.0	4000 ± 156	5 ± 0.2
100 ED-20 + 40 ORPP + 75 ocher	102 ± 4.1	10163 ± 350	156 ± 5.8	45 ± 2.3	4110 ± 160	9 ± 0.4
100 ED-20 + 40 ORPP + 100 ocher	55 ± 2.0	12120 ± 445	95 ± 4.0	32 ± 1.6	4860 ± 184	4 ± 0.2

Note: Gben—bending stress; Eben—modulus of elasticity in bending; Gcom—compressive strength; Gten—tensile strength; Eten is the tensile modulus of elasticity; aim—impact strength.

**Table 3:** Values of the curing process of epoxy composites.

Composition, Parts by Mass, Cured by 15 Parts by Mass of PEPA	τ_gel_, Min	τ_cur_, Min	T_max_, °C
100ED-20 + 40ORPP	27	38	88
100ED-20+40ORPP+0.5 ocher	21	30	99
100ED-20+40ORPP +75 ocher	17	29	96

Note: τ_gel_ is the duration of gelation process, τ_cur_ is the duration of curing, T_max_ is the maximum temperature of the sample self-heating during curing.

**Table 4:** Physico-chemical properties of epoxy composites.

Composition, Parts by Mass, Cured by 15 Parts by Mass of PEPA	T_in_, °C	T_f_ °C	Yield of CS at T_f_,% Mass	T_v_, °C	OI, % vol.
100ED-20	200	390	40 (390 °C)	86	19
100ED-20 + 40ORPP	230	370	54 (370 °C)	132	28
100ED-20 + 40ORPP + 0.5 ocher	240	360	58 (360 °C)	148	28
100ED-20 + 40ORPP + 50 ocher	242	370	69 (370 °C)	170	30
100ED-20 + 40ORPP + 75 ocher	245	370	72 (370 °C)	190	31
100ED-20 + 40ORPP + 100 ocher	245	370	76 (370 °C)	210	32

Note: T_in_, T_f_—initial and final temperature of the main stage of thermolysis; CS—carbonized structures, T_v_—Vicat heat resistance, OI—oxygen index.

**Table 5:** Properties of epoxy composites.

Parameters of Microwave Modification of Composition in parts by Mass: 100ED-20 + 40ORPP + 0.5 Ocher + 15PEPA	G_ben_, MPa	E_ben_, MPa	G_t_, MPa	E_t_, MPa	a_im_, kJ/m^2^
Without microwave modification	105 ± 4.2	3500 ± 140	58 ± 2.6	2317 ± 92	11 ± 0.5
200 W, 15 s	108 ± 4.3	4192 ± 165	57 ± 2.3	2804 ± 112	11 ± 0.5
200 W, 30 s	128 ±4.5	4438 ± 168	63 ± 2.8	2889 ± 115	13 ± 0.6
200 W, 45 s	100 ± 4.0	4101 ± 145	55 ± 2.0	2039 ± 81	7 ± 0.4
250 W, 30 s	130 ± 4.5	4892 ± 175	59 ±2.4	3162 ± 125	13 ±0.6
300 W, 30 s	132 ± 4.6	3861 ± 145	75 ± 3.0	2674 ± 110	12 ± 0.5
350 W, 30 s	158 ± 6.0	5193 ± 200	75 ± 3.0	3075 ± 123	15 ± 0.7
400 W, 30 s	121 ± 4.8	3685 ± 142	58 ± 2.3	2628 ± 105	10 ± 0.5
500 W, 30 s	115 ± 4.6	3470 ± 135	45 ± 1.8	2031 ± 81	8 ± 0.4

Note: G_ben_—bending stress; E_be_—modulus of elasticity in bending; G_t_—tensile strength; E_t_—tensile modulus of elasticity; a_im_—impact strength.
